# Bioelectrical Impedance Analysis-Derived Phase Angle and Body Composition Are Predictors of Health-Related Fitness in Children and Adolescents with Obesity

**DOI:** 10.3390/children9121943

**Published:** 2022-12-11

**Authors:** Giada Ballarin, Maria Rosaria Licenziati, Paola Alicante, Olivia Di Vincenzo, Giuliana Valerio, Luca Scalfi

**Affiliations:** 1 Department of Movement Sciences and Wellbeing, University of Naples “Parthenope”, 80133 Naples, Italy; 2 Obesity and Endocrine Disease Unit, Department of Neurosciences, Santobono-Pausilipon Children’s Hospital, 80129 Naples, Italy; 3 Department of Public Health, Federico II University of Naples, 80131 Naples, Italy

**Keywords:** obesity, health-related physical fitness, cardiorespiratory fitness, strength, childhood

## Abstract

There is little evidence in children and adolescents with obesity of the relationships between muscle strength/cardiorespiratory fitness (both components of health-related fitness = HRF) and body composition. Body composition and HRF were studied in 281 children and adolescents with obesity to explore their mutual relationship and to identify the predictors of HRF. By performing a bioelectrical impedance analysis (BIA), the fat-free mass (FFM) and percentage of body fat (%BF) were calculated, and the phase angle (PhA) was recorded. Handgrip strength (HGS), the standard broad jump (SBJ), and five broad jumps (FIVEBJ) were considered for the assessment of muscle strength, and the six-minute walking distance (SIXMWD) for cardiorespiratory fitness. The BMI Z-score was slightly higher in boys, and the %BF was higher in girls, with no difference in the FFM. HGS, the SBJ, and FIVEBJ were greater in the male sex. After controlling for sex, HGS was associated with the FFM, and with height, weight, and absolute BMI. On the contrary, the SBJ and FIVEBJ were negatively associated with adiposity, with a weak relationship with the FFM. The SIXMWD was only poorly related to height, the BMI Z-score, and the waist-to-height ratio. These results were confirmed with a multiple regression analysis. HGS, the SBJ, and FIVEBJ were higher in the first compared to the third tertile of the PhA in both sexes. The PhA also remained a consistent predictor of HGS, the SBJ, and FIVEBJ in a multiple regression analysis. In conclusion, the following predictors have been identified for HRF: the FFM for the isometric strength of the upper limbs and adiposity indicators for the SBJ and FIVEBJ. The PhA emerged as a proxy index of muscle strength.

## 1. Introduction

Health-related fitness (HRF), which is a theoretical construct that defines the ability of performing physical activities positively related to health status and well-being, includes different components, such as muscular strength, muscular endurance, cardiorespiratory endurance, flexibility, and body composition [1]. Parallel to changes in body weight and body composition, age-dependent variations of HRF are observed in children/adolescents; actually, in the first two decades of life, muscular strength and cardiorespiratory fitness have been associated with health status and well-being [2,3,4,5].

The components of HRF can be assessed using different procedures/tests, sometimes combined in so-called fitness batteries; handgrip strength (HGS) and the standing broad jump (SBJ) are commonly used as indexes of muscular strength [6], and the six-minute walking distance (SIXMWD) is commonly used as an index of cardiorespiratory fitness [4].

In the general pediatric population, fat-free mass (FFM) is a positive determinant of muscle strength [7,8], while an inverse association is observed between muscle strength and body mass index (BMI, as an absolute value or a Z-score) as well as waist circumference (WC) [2,9,10,11]. HRF is impaired in children/adolescents with obesity compared to their normal-weight counterparts [12,13,14,15,16] along with reduced physical activity and poorer scores in the physical aspects of quality of life, and it is associated with lower cardiometabolic parameters in later life [13,17,18,19]. Furthermore, previous studies have consistently shown that a lower cardiorespiratory fitness may be considered an additional trait of cardiometabolic risk clustering in youths since it is associated with insulin resistance, a higher blood pressure, dyslipidemia, dysglycemia, and an increased arterial stiffness as well as predicted mortality in adults [4,20]. Less robust evidence has indicated that musculoskeletal fitness in children/adolescents is also positively associated with obesity indicators (BMI, WC) and with a lower risk related to the clustered variables of cardiometabolic risk [21].

In children/adolescents with excess body fat, muscle strength has been positively associated with maturity status and the FFM [22,23], and it has been negatively associated with the percentage of body fat (%BF) and abdominal obesity [11,23]; in addition, the %BF and WC were found to be negative predictors of the SBJ and the sit-to-stand test [10,11]. Thus, in nutritional surveillance and clinical nutrition, much interest is expected to be focused on the combined evaluation of body composition and HRF. In prevention and clinical nutrition, bioelectrical impedance analyses (BIA) are widely used as a field technique for the estimation of body composition in terms of the FFM, body cell mass (BCM), and total body water (TBW). As an alternative, the phase angle (PhA), which is a directly measured raw BIA variable, is suggested to be a proxy index of BCM, the distribution between the extracellular water (ECW) and TBW, and cellular integrity [24]. The PhA increases progressively over the first two decades of life, markedly in teenagers (especially in the male sex), with no definite evidence regarding the effect of overweight/obesity both in youths and in adults [24,25,26]. In addition, the PhA was found to be a good marker of the quality of appendicular muscle mass [27] and has been related to prognostic factors and clinical outcomes in critically ill, HIV-infected, and hospitalized pediatric patients [28,29,30]. Interestingly, an association between musculoskeletal fitness and raw BIA variables has been observed in normal-weight children/adolescents as well as in healthy young adults [7,31,32].

Against this background, the present study was carried out in children/adolescents with obesity (1) to evaluate body composition, raw BIA variables, and HRF; (2) to explore the relationships between body composition, raw BIA variables, and HRF; and (3) to identify the predictors of HRF among the anthropometric and body composition variables, with a particular interest in the PhA.

## 2. Materials and Methods

This was a cross-sectional study performed from 1 April 2017 to 31 December 2017. Two hundred eighty-eight children and adolescents with obesity were admitted for their first visit to the Obesity Unity of Santobono Pausilipon Children’s Hospital in Naples, Italy. The inclusion criteria for consideration for this study were as follows: age 8–14 years and BMI ≥ 97th percentile [33]. Meanwhile, the exclusion criteria were cognitive or physical limitations to undergoing physical fitness tests. Finally, two hundred eighty-one children and adolescents (53.4% male sex, mean age of 10.8, age range of 7–16 years) were effectively enrolled in this cross-sectional study. Before their enrolment, children and/or their parents or legal guardians accepted to sign the written informed consent for all procedures. This study was approved by the Institutional Review Board of the Ospedale Cardarelli, Naples (reference number 33/28.03.2017).

### 2.1. Anthropometric Measurements

The participants were asked to wear only light clothes. Height, weight, and WC were measured by the same investigator. It was recommended to avoid high-intensity physical exercise in the 12 h before the test. Body weight and height were measured to the nearest 0.1 kg and 0.5 cm, respectively, with a mechanical column scale and a stadiometer (Seca 711 and Seca 220, respectively; Seca, Hamburg, Germany). BMI was derived as weight/height^2^ (kg/m^2^). BMI Z-scores were calculated according to the WHO growth reference [33]. WC was measured with the participant in a standing position with a flexible tape and was taken midway between the 10th rib and the iliac crest. Waist-to-height ratio (WHR) was calculated as the ratio of WC to height.

### 2.2. Bioelectrical Impedance Analysis

BIA was performed on both sides of the body (Human Im Touch analyzer; DS Medica, Milan, Italy). PhA was measured at 50 kHz in standardized conditions: ambient temperature of 23–25°C, fasting > 3 h, empty bladder, and supine position for at least 10 min before starting the measurement. Participants were asked to lie down without any contact between the limbs and trunk. A standard tetrapolar technique was used: injector electrodes were placed in the metacarpophalangeal and metatarsophalangeal joints, while the sensing electrodes are usually placed at the end of the superior and inferior limbs. The mean value of right and left sides of the body was considered for statistical analysis. FFM was estimated from impedance at 50 kHz using a BIA equation developed for children/adolescents with obesity [34]. Fat mass (FM) was calculated as body weight minus FFM, and percentage of body fat (%BF) was calculated as the ratio of FM to body weight.

### 2.3. Physical Fitness Assessment

Selected HRF tests were assessed according to standard procedures.

(1) HGS (in kg) was measured using a Dynex dynamometer (MD Systems Inc., Westerville, OH, USA) to assess the isometric strength of both arms. A pretest was done to allow the subject to become familiar with the instrument. Participants were asked to stand upright with their shoulder adducted and neutrally rotated, the elbow fully extended, and the forearm and wrist neutrally positioned. They performed three maximal contractions (each lasting 5 s) with each hand, with a 1 min rest between tests [35]. Maximum HGS was finally defined as the highest value of the six attempts (three for the dominant and three for the nondominant arm).
(2) SBJ (in centimeters) was used to assess lower-body muscle strength [5]. Participants performed a two-foot take-off and landing (swinging of the arms and flexing of the knees allowed). Participants performed two attempts to jump as far as possible while landing on both feet and without falling backwards. Length was measured to the nearest point of contact. The best value was used for statistical analysis.
(3) Five broad jump (FIVEBJ, in centimeters) was used to assess lower-body muscle strength and power. Participants performed a two-foot take-off and landing five times with swinging of the arms and flexing of the knees permitted to provide forward drive. Length was measured to the nearest point of contact of the landing at the end of the five jumps. The best value of two attempts was used for analysis.
(4) Six-minute walking test (in meters) was performed indoors [36] along a corridor (length of 20 m) that was marked every 2 m with a brightly colored tape. Two cones were used to indicate the beginning and the end points of the walking course. Each participant was tested individually and was asked to walk the longest distance possible in 6 min. Only standardized phrases for encouragement (e.g., “keep going” and “you are doing well”) and announcement of time remaining were given.

### 2.4. Statistical Analysis

We expressed results as mean ± standard deviation or percentage (where opportune), and predetermined statistical significance was *p* < 0.05. All statistical analyses were performed using the Statistical Package for Social Sciences (SPSS Inc, Chicago, IL, USA) version 26. The Shapiro–Wilks normality test was applied to assess the normality of data distribution: nonparametric variables were logarithmically transformed to perform the analysis, but they were expressed as untransformed values for clarity of interpretation. Differences between groups were assessed using one-way ANOVA or general linear model (when data were adjusted for age). Nonpartial correlation analysis was used to assess correlation between variables of interest. The stepwise regression was used to assess predictors of HRF components, such as demographic, anthropometric, body composition, and BIA variables.

## 3. Results

The general characteristics of the study group (children/adolescents with obesity) were reported by sex in Table 1. No statistical differences emerged for weight and height, while the BMI Z-score was slightly higher in boys compared to girls. As expected, girls showed a greater %BF, while no significant differences between the sexes were found for the FFM or FFMI (+6% and +3%, respectively). The PhA varied from 3.7 to 6.4 degrees in boys and from 3.9 to 6.3 degrees in girls (no significant difference). HGS (+8%), the SBJ (+13%), and FIVEBJ (+12%) were greater in boys than in girls, while the SIXMWD was very similar between the sexes. After controlling for sex, HGS moderately correlated with the SBJ (r = 0.350) and FIVEBJ (r = 0.338), and it weakly correlated with the SIXMWD (0.187) (data not shown).

**Figure 1:**
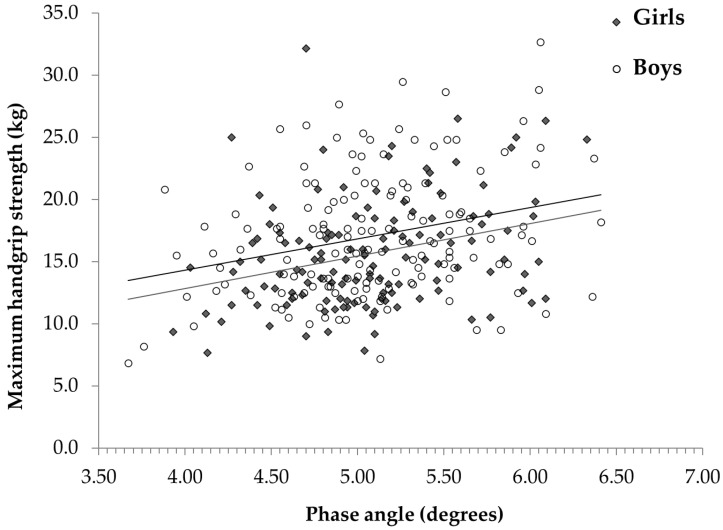
Relationship between maximum handgrip strength and phase angle at 50 kHz in boys and girls.

**Figure 2:**
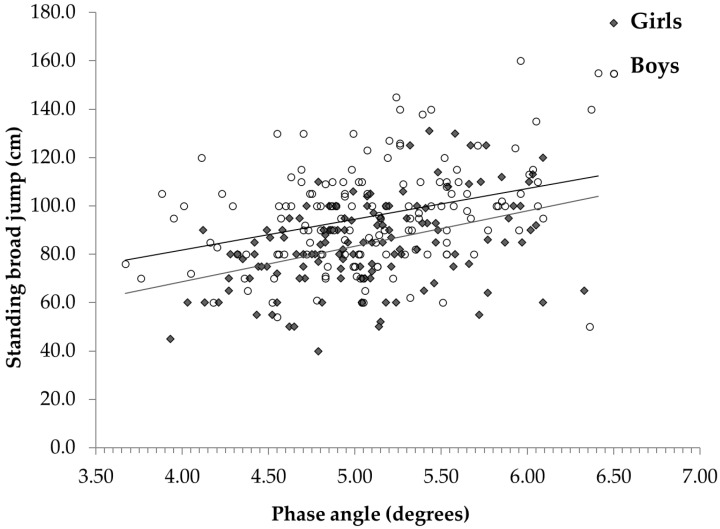
Relationship between standing broad jump and phase angle at 50 kHz in boys and girls.

Table 2 shows the relationships between HRF and the anthropometric/body composition variables in the children/adolescents with obesity studied in the present study. HGS was associated with most of the parameters, the FFM in particular, whereas the SBJ and FIVEBJ were negatively associated with the BMI Z-score and WC and were even more strictly associated with the %BF (Table 2). The SIXMWD was inversely and weakly correlated with weight, the BMI Z-score, and the WC, but it was not associated with body composition. As for the raw BIA variables, there was a significant relationship between HGS/jumps and the PhA, while there was no association with the SIXMWD (Figure 1 and Figure 2 and Table 2).

Figure 1 and Figure 2 show the relationships between HGS/SBJ and the PhA in children/adolescents with obesity. Although there was a difference between the sexes, the slopes of the regression lines were similar in boys and girls. The values of the HRF tests according to the tertiles of the PhA (T1, T2, and T3) are reported in Table 3. HGS was slightly higher, but not significant in T3 compared to T1 in boys (+9.9%), while a more marked difference was found in girls (+13.4%). The SBJ and FIVEBJ statistically differed between T3 and T1 both in boys (+13.9 and +12.5 cm, respectively) and between T3 and T1 in girls (+20.9 and +16.5 cm, respectively).

Finally, a multiple regression analysis was performed to identify the determinants of the HRF tests. The first model included the anthropometric variables (Table 4); in addition to sex and age, weight and height emerged as significant predictors of HGS, but only the BMI Z-score was significant for the SBJ and the FIVEBJ. Height and the BMI Z-score were independent predictors of SIXMWD.

The predictivity of the model increased when the FFM, %BF, and PhA were added to the aforementioned variables. After controlling for sex and age, HGS was related to the FFM, while the %BF and WHR were predictors of the SBJ. Interestingly, the PhA was found to be directly and consistently related to all the tests (Table 4). The SIXMWD was directly associated with height and the BMI Z-score, but the R squared was low.

## 4. Discussion

The present study focused on the relationships between HRF and body composition (with a specific interest in the BIA-derived PhA) in children/adolescents with obesity. In a multivariate analysis, the PhA was found to be an independent predictor of HGS, the SBJ, and FIVEBJ but not the SIXMWD. The FFM emerged as a significant predictor of HGS, while the %BF was a negative predictor of the jump tests. Height and the BMI Z-score were identified as weak predictors of the SIXMWD.

HRF in the first two decades of life is associated with cardiovascular risk, future health status, and well-being [2,4,5]. As for excess body fat, musculoskeletal strength and cardiorespiratory fitness are impaired in children/adolescents with obesity compared to their normal-weight counterparts [12,13,14,15], with reduced physical efficiency and physical activity, poorer scores in the physical and psychological domains of quality of life, and altered cardiometabolic parameters in later life [17,18]. Surprisingly, in the general pediatric population, there are limited studies focusing on the relationships between musculoskeletal strength/cardiorespiratory fitness and body composition (another component of HRF); for instance, muscle strength has been related to the FFM [7,8] and also to the BIA-derived PhA [7,25], which is thought to be a proxy index of the BCM and the ratio between the ECW and TBW. In children/adolescents with excess body fat, muscle strength has been directly associated with maturity status and the FFM [22,23], and it has been negatively associated with the %BF and abdominal obesity [11,23]. Against this background, this paper aimed to appraise the relationship between HRF and body composition (assessed using a BIA) in children/adolescents with obesity. Given the range of field-based HRF tests, HGS and the SBJ (plus FIVEBJ) were selected to assess muscle strength, and the SIXMWD was selected to evaluate cardiorespiratory fitness.

As for muscle strength, previous studies have shown that HGS is higher in absolute values in children/adolescents with excess body fat compared to their normal-weight counterparts, but it is lower when scaled to weight [11,22,23]. In line with a previous study [11], the results presented here showed that HGS significantly correlated with several anthropometric and body composition variables. In a multiple regression analysis including anthropometric variables only (Table 4), sex, age, weight, and height were independent predictors of HGS (R squared = 0.507), with no significant effect in the BMI Z-score or the WHR. When body composition variables were also included, age, the FFM, and the PhA (but not sex, age, weight, height, the WHR, or the %BF) were identified as independent predictors of HGS, accounting for more than 50% of the total variance, a figure greater than that which was previously reported [11]. Thus, our findings gave more detailed information on the determinants of HGS in children/adolescents with obesity, which were in partial disagreement with the previous suggestions of an inverse relationship with the %BF and WC [11,23]. Remarkably, in line with the evidence for the general pediatric population [7,25,31], the PhA came out as an independent predictor of HRF, suggesting that this raw BIA variable may be helpful in interpreting the variability of muscle strength and in giving more information on muscle function.

The SBJ is frequently included in fitness batteries for youths as a measure of the explosive power of the lower limbs [5]. The relationships between the SBJ and body composition have previously been evaluated by few papers regarding healthy children/adolescents or those practicing sports, which have shown a positive association with the FFM and a negative association with the BMI Z-score, WHR, and %BF [8,10,11]. In addition, the SBJ is lower in children/adolescents with obesity compared to their normal-weight counterparts, once again with a positive relationship with the FFM and a negative one with the %BF [11]. Our results confirmed that the SBJ significantly and inversely correlated with the BMI Z-score, %BF, and WHR but showed only a weak association with the FFM. Interestingly, a higher correlation coefficient was observed between the SBJ and the PhA. These findings were confirmed with a multivariate analysis, showing that the WHR and %BF along with the PhA accounted for 37% of the total variance; the predictivity was much lower (24% of variance) when the model included anthropometric variables only. As an additional approach, a FIVEBJ test was performed to measure the horizontal and vertical power of the lower limbs (results also affected by balance and coordination). As a new finding, moderate to strong correlations were found between FIVEBJ and the different anthropometric and body composition variables: sex, age, the WHR, the BMI Z-score, and the FFM were all predictors of FIVEBJ.

Overall, in agreement with the preliminary results for adolescent athletes [37], for the first time, this study demonstrated that the PhA was an independent predictor of muscle strength in children/adolescents with obesity. The PhA significantly correlated with the HRF tests and was found to be an independent predictor of HRF in multiple regression analyses. Given its role as a proxy marker of the skeletal muscle mass and muscle function [27], the PhA may be taken into consideration in the clinical practice; unfortunately, there is still scarce evidence of its relationship with clinical outcomes, with there only being evidence for severe pediatric conditions [28,29,30]. Thus, our findings suggested the importance of developing normative values for raw BIA variables in pediatric populations and figuring out their predictive efficiency. In our view, the PhA might be used as additional information for the assessment of muscle structure rather than for the prediction of strength and for the interpretation of the HRF test in a more appropriate way.

Finally, it is worth mentioning that the SIXMWD is extensively measured in the general pediatric population as well as in adults and the elderly to assess cardiovascular fitness (submaximal exercise). Different equations for the prediction of the SIXMWD have been developed for the pediatric population, with a direct relationship with age and height and an inverse association with the BMI [38,39,40]. Recently, a predictive equation was developed for children/adolescents with obesity that includes the BMI and WC (negative predictors) in addition to age and height and that explains 70% of the variability in both sexes [41]. In this study, our predictive models showed no effect of weight, the BMI Z-score, or the %BF on the SIXMWD probably because of a less heterogeneous sample compared to those of previous studies. Indeed, a great discrepancy was found regarding the SIXMWD among published papers (including the present one), without any obvious explanation [38,39,41].

The present study presented some limitations: only children/adolescents with obesity were included with no comparison with their normal-weight counterparts; it was a single-center study; the sample size did not allow the running of analyses in different age groups; the cross-sectional design did not allow the analysis of changes due to weight loss; and body composition was assessed using BIA, a well-known and reliable technique, but there was no comparison with reference methods (indeed, the latter were quite difficult to apply in such an age range). This study also presented several strengths: The physical fitness tests selected for the domains of cardiorespiratory fitness and strength are supported by a wide literature due to their features of high feasibility and reproducibility [5]. Measurements were performed by the same operator, and the relationships between HRF and the potential predictors were explored in a systematic way (using univariate and multivariate regression models).

## 5. Conclusions

In conclusion, this study provided some additional information on muscle strength, the BIA-derived PhA, body composition, and their mutual relationships in children/adolescents with obesity, suggesting that the combination of these variables may be useful for assessing nutritional status and HRF in the pediatric population. The PhA emerged as an independent predictor of HGS, the SBJ, and FIVEBJ, confirming that this raw BIA variable might play a role in a more appropriate evaluation of HRF in the clinical practice.

## Figures and Tables

**Table 1:** General characteristics, body composition, BIA-derived phase angle, and physical fitness of the whole study group.

	Boys (n = 150)	Girls (n = 131)	*p*
Age (yrs)	11.0 (1.8)	10.7 (1.9)	0.197
Weight (kg)	63.9 (16.5)	63.4 (17.3)	0.793
Weight Z-score	2.83 (0.91)	2.72 (0.96)	0.318
Height (cm)	148.1 (11.2)	145.9 (10.7)	0.097
Height Z-score	0.70 (1.02)	0.55 (1.02)	0.243
Body mass index—BMI (kg/m^2^)	28.8 (4.0)	29.2 (4.8)	0.097
BMI Z-score	3.3 (0.8)	3.0 (0.8)	0.005
Waist circumference—WC (cm)	97.4 (11.3)	97.0 (12.1)	0.777
WC/height—WCH	0.66 (0.05)	0.67 (0.07)	0.344
Fat-free mass—FFM (kg)	40.4 (9.8)	38.1 (9.7)	0.053
FFM index—FFMI (kg/m^2^)	18.2 (2.3)	17.7 (2.5)	0.145
Percentage of body fat (%)	36.6 (7.4)	38.7 (7.5)	0.015
Phase angle (degrees)	5.09 (0.55)	5.08 (0.54)	0.961
Handgrip strength (kg)	17.1 (4.9)	15.8 (4.4)	0.015
Standing broad jump (cm)	95.8 (20.6)	84.0 (19.4)	< 0.001
Five broad jumps (cm)	473.0 (86.5)	424.1 (80.0)	< 0.001
Six-minute walking distance (m)	461 (45)	457 (45)	0.501

Data are expressed as mean (standard deviation) or number (percentages) where appropriate.

**Table 2:** Partial correlations (adjusted for sex and age) between health-related physical fitness (HRF) tests and height, weight, BMI, and body composition.

HRF Tests	Height	Weight	BMI	BMI Z-Score	WHR	FFM	FFMI	%BF	PhA
Handgrip strength	0.684 *	0.661 *	0.453 *	−0.086	0.084	0.720 *	0.535 *	−0.036	0.285 *
Standing broad jump	0.170 *	−0.020	−0.194 *	−0.366 *	−0.343 *	0.143 *	0.075	−0.327 *	0.361 *
Five broad jumps	0.163 *	−0.001	−0.150	−0.318 *	−0.293 *	0.156 *	0.103	−0.302 *	0.355 *
Six-minute walking distance	0.160 *	0.062	−0.059	−0.214 *	−0.155 *	0.063	−0.072	−0.007	0.074

BMI = body mass index; FFM = fat-free mass; FFMI = fat-free mass index; %BF = percentage of body fat; PhA = phase angle at 50 kHz (whole body); and * = *p* < 0.001.

**Table 3:** Health-related fitness tests expressed for tertiles of phase angle in boys and girls.

Tertiles of Phase Angle						
	Boys			Girls		
	1st	2nd	3rd	1st	2nd	3rd
Handgrip test (kg)	16.1 (4.7)	17.4 (4.9)	17.7 (5.3)	15.1 (4.7) ^a^	14.8 (3.6) ^b^	17.1 (4.5) ^a,b^
Standing broad jump (cm)	89.7 (17.6) ^a^	95.0 (20.6)	102.2 (22.5) ^a^	75.9 (14.2)	85.1 (16.5) ^a^	91.8 (20.1) ^a,b^
Five broad jumps (cm)	445.5 (74.0) ^a^	473.0 (85.6)	501.3 (92.9) ^a^	391.9 (62.9)	418.7 (68.2)	456.4 (91.1) ^a,b^
Six-minute walking distance (m)	464 (43)	466 (46)	461 (44)	454 (39)	458 (47)	458 (46)

Data (adjusted for age) are expressed as mean (standard deviation). ^a^ *p* < 0.05 vs. 1°tertile; ^b^ *p* < 0.05 vs. 2°tertile.

**Table 4:** Predictors of health-related physical fitness in the whole study group taking into consideration demographic and anthropometric variables only (Model 1) or body composition and BIA variables (Model 2).

		Sex	Age	Weight	Height	WHR	BMI Z-Score	FFM	%BF	PhA	R^2^
Handgrip strength	Model 1	0.094 *	0.241 **	0.252 **	0.116 **	NS	NS	-	-	-	0.507
	Model 2	NS	0.258 **	NS	NS	NS	NS	0.504 **	NS	0.150 **	0.568
Standing broad jump	Model 1	0.234 **	0.266 **	NS	NS	−0.341 **	NS	-	-	-	0.242
	Model 2	0.214 **	0.236 **	NS	NS	−0.242 **	NS	NS	−0.171 **	0.273 **	0.349
Five broad jumps	Model 1	0.260 **	0.235 **	NS	NS	−0.290 **	NS	-	-	-	0.208
	Model 2	0.346 **	NS	NS	NS	NS	−0.364 **	0.119 *	NS	0.336 **	0.331
Six-minute walking distance	Model 1	NS	NS	NS	0.149 *	NS	−0.154 *	-	-	-	0.046
	Model 2	NS	NS	NS	0.149 *	NS	−0.154 *	NS	NS	NS	0.046

WHR = waist-to-height ratio; BMI = body mass index; FFM = fat-free mass; %BF = percentage of body fat; PhA = phase angle; R^2^ = adjusted R squared; * *p* < 0.05; ** *p* < 0.01; and NS = not significant.

## Data Availability

Not applicable.
